# Caffeine Improves Left Hemisphere Processing of Positive Words

**DOI:** 10.1371/journal.pone.0048487

**Published:** 2012-11-07

**Authors:** Lars Kuchinke, Vanessa Lux

**Affiliations:** Department of Psychology, Ruhr University Bochum, Bochum, Germany; University of Pittsburgh, United States of America

## Abstract

A positivity advantage is known in emotional word recognition in that positive words are consistently processed faster and with fewer errors compared to emotionally neutral words. A similar advantage is not evident for negative words. Results of divided visual field studies, where stimuli are presented in either the left or right visual field and are initially processed by the contra-lateral brain hemisphere, point to a specificity of the language-dominant left hemisphere. The present study examined this effect by showing that the intake of caffeine further enhanced the recognition performance of positive, but not negative or neutral stimuli compared to a placebo control group. Because this effect was only present in the right visual field/left hemisphere condition, and based on the close link between caffeine intake and dopaminergic transmission, this result points to a dopaminergic explanation of the positivity advantage in emotional word recognition.

## Introduction

An important dimension to categorize emotional content is valence, i.e. how positively or negatively an event or an object is evaluated. It has often been shown that the valence dimension affects human performance, but the direction of these valence effects seem to be inconsistent at a first glance: Whereas both, positive and negative, valences enhance recognition memory for pictures [1], sounds [2] or words [3], [4] comparably well (though see [5], [6]), the two valences have opposing effects in most tasks that require simple and fast identifications of, or decisions on, emotional target stimuli (e.g., [7]). Positive valence seems to be beneficial for solving these types of tasks, whereas negative valence slows down performance. Thus, for example, in face processing, a happy-face-advantage [8], [9] describes an identification advantage “both prior to …(or) during overt attentional processing” [9]. One interpretation of this effect is that of a bias, caused by the higher familiarity shared by happy faces that are encountered more often in everyday situations [9].

In emotional word recognition a consistent processing advantage is observed for positive words [10]–[13]. In addition, negative words are often observed to be processed comparably slow as or even slower than neutral words [10], [13], [14]. In contrast to face processing, familiarity cannot possibly explain this positivity advantage. In word recognition the stimulus material is well controlled for familiarity (or word frequency as an objective measure of familiarity) across the different emotional word conditions. Similar results are also known from the affective Stroop task, where color naming is slower for negative compared to neutral words [15]–[18]. This effect of negative words is known to be modulated by other lexical or affective features, like higher word frequency [19], [20] and/or high emotional arousal [14], [21], that reduce or sometimes even reverse the effects of negative valence (see [22] for recent review). In general, the slowdown in processing of negatively valenced stimuli has been attributed to automatic vigilance [7], [23], [24], i.e. the hypothesis that negative stimuli are attended to preferentially. As a consequence, attention may be disengaged more slowly from negative compared to positive and neutral stimuli [25], leading to the observed disadvantage in simple or speeded cognitive tasks wherein valence is not task-relevant [24].

The higher attractiveness of negative stimuli might explain the disadvantage of negative items compared to neutral ones in implicit and fast recognition paradigms and it also explains their advantage in recognition memory - but it doesn’t help to understand the described positivity advantage. The same advantage also been documented for verbal working memory: Here, a link between verbal processing and positive emotions has been reported by Gray [26] who observed that a positive affective state enhanced verbal working memory performance whereas it was impaired following a negative state. Interestingly, the opposite pattern was revealed for spatial working memory performance that was found to be enhanced by negative states and impaired by positive states.

In case of verbal processing the positivity advantage seems to point to a specificity of the left hemisphere (LH): In almost all right-handed and most left-handed individuals language is left lateralized [27]. An advantage of positive words might therefore be related to left hemisphere functioning. But language lateralization alone cannot explain the positive word advantage. Using a Divided Visual Field Paradigm, Holtegraves and Felton [28] observed that positive words were recognized faster when presented to the right visual field/left hemisphere (RVF/LH) than when they were presented to the left visual field/right hemisphere (LVF/RH). This laterality effect was significantly smaller for neutral words and not present in negative words. The asymmetry for positive words must therefore best be explained by a combination of both, their emotional content and a LH superiority in word recognition [28].

Hemispheric asymmetries in emotion processing in general have been supported by two opposing models. The valence hypothesis model (VHM) proposes an association of approach-related/positive valence with LH processing and of withdrawal-related/negative valence with RH processing in emotion experience and expression [29]–[31]. The empirical data sometimes also support a RH model that associates the perception of both valences with RH functioning (for reviews see [32], [33]). Given the observed link between left hemispheric processing and positive valence it is evident that the positivity advantage observed in emotional word recognition is more in agreement with the predictions of the VHM. The proposed brain basis of the VHM is a right frontal behavior inhibition system linked to withdrawal and negative emotion [34] and a left-lateralized (orbito-)frontal reward system [31], [35] in cooperation with the mesolimbic dopaminergic system that is linked to approach behavior and positive affect [30]. In particular, the link to the mesolimbic dopaminergic system for the processing of positive information has gained little attention so far in the literature and direct empirical evidence in support of this hypothesis is lacking. This is surprising given that it is generally agreed that reward processing is linked to the functioning of the fronto-striatal dopaminergic system [36]–[38]. Moreover, evidence in favor of a left-biased hemispheric asymmetry has been discussed in the literature on the striatal dopaminergic system [39]–[41] which could further help to explain this relationship.

The present study examined the hypothesis of a link between the positivity advantage and dopaminergic transmission by the administration of caffeine in a divided visual field emotional word recognition study. Caffeine is a psychoactive substance that in low doses blocks the inhibitory adenosine receptors in the brain, thereby functioning as an adenosine antagonist. This antagonist behavior leads to an increase in central nervous activity most probably via an increased dopaminergic transmission due to multiple interactions with dopamine receptors in dopamine-rich brain regions [42]–[44]. At the behavioral level, caffeine consumption at a normal dose leads to faster responses and fewer errors in simple cognitive tasks [45]–[50], but also to improvements in conflict monitoring and task switching [51], [52]. It is discussed that these caffeine effects result from increases in arousal levels and wakefulness in cognitive processing [44].

Mood effects of caffeine consumption are also reported, with low doses of caffeine heightening subjectively reported positive mood [48], [53], [54]. On the other hand, besides these mood effects no further evidence for a modulation of emotional processing in humans is discussed in the literature. Instead, caffeine effects on human behavior have mainly been related to a speeding up of psychomotor functioning [42] that is based on dopaminergic effects in the striatum (e.g. [55]). Hence, improved sensorimotor gating, which is known to depend on striatal functioning, might be a primary locus of the ‘cognitive’ effects of caffeine in humans [42]. We propose a slightly different interpretation here, an effect of caffeine that interacts with the emotional valence of the stimuli: If the hypothesis of a link between the LH positivity advantage in emotional word recognition and the dopaminergic system is true, an effect of caffeine administration is expected to modulate word recognition by specifically enhancing the processing of positive words. Moreover, this effect of caffeine should mainly be evident in words presented in the RVF, since these are initially processed in the language-dominant LH.

## Methods

The experiment was conducted in accordance with the Declaration of Helsinki and approved by the local ethics committee of the Faculty of Psychology, Ruhr-University Bochum. Written informed consent was obtained from all participants.

### Participants

Sixty-six healthy participants age 24.3 years (19–32 years) were randomly assigned to either a caffeine group (n = 33, 9 males) or a placebo control group (n = 33, 12 males). All were right handed according to the Edinburgh Handedness Inventory [56] (range 60–100), and had normal or corrected-to-normal vision, reported no history of a neurologic or psychiatric disorder and normal caffeine consumption (on average 1.58 cups a day, range 0–8 cups). None of the participants had consumed caffeine, nicotine or alcohol within the 12 h prior to the experiment.

### Materials and Procedure

Emotional words were selected from the Berlin Affective Word List [57] and a 3 (EMOTION)*2 (LEXICALITY) design was employed in analogy to the signal-detection approach described in [58]. Applying signal detection theory allows for the computation of hit (HIT) and false alarm (FA) rates, as well as measures of performance P (word-pseudoword discriminablity) and response bias B for each emotion condition (see [58]). Six stimulus lists of 50 stimuli each were used, three lists contained emotional words (positive, neutral or negative) and three lists consisted of emotional pseudowords (positive, neutral or negative). In contrast to Windmann and colleagues [58], who build pseudowords by interchanging vowels within emotional words, all pseudowords in the present study were so-called pseudohomophones, i.e. pseudowords that differ from real words in orthography but not in phonology (e.g. ‘BRANE’). The six lists of 4–8 letter words and pseudowords were carefully matched for factors known to affect lexical decision performance. Accordingly, the three emotional word lists and the three pseudoword lists did not differ in number of letters, word frequency, number of phonemes, number of syllables and number of orthographic neighbors. Furthermore the three lists of emotional words were matched for arousal. Pseudowords differed slightly in their arousal values, with negative pseudowords having higher values compared to positive and neutral pseudowords, whereas the latter did not differ (see Table 1).

**Table 1:** Word statistics.

	words					pseudowords				
	neg	neu	pos	f	p-value	neg	neu	pos	f	p-value
**valence**	−1.57	−0.04	+1.55	1293.71	**<0.001**	−1.61	0.11	+1.60	1082.81	**<0.001**
**arousal**	3.18	3.04	3.13	1.10	0.336	3.65	2.45	2.36	177.55	**<0.001**
**imageability**	4.00	4.23	4.23	0.488	0.615	4.06	4.09	3.98	0.088	0.916
**letters**	6.40	6.26	6.26	0.221	0.802	6.60	6.46	6.62	0.329	0.721
**phonems**	5.56	5.56	5.58	0.005	0.995	5.76	5.58	5.92	1.162	0.316
**syllables**	2.14	2.10	2.20	0.298	0.743	2.14	2.18	2.24	0.510	0.602
**frequency**	5.97	6.72	7.94	0.461	0.631	11.42	12.14	10.24	0.395	0.674
**ortho n**	0.86	1.02	1.02	0.221	0.802	0.76	0.88	0.80	0.109	0.897

ortho n  =  number of orthographic neighbors.

f  =  ANOVA f-value.

A single-blind, placebo-controlled design was used. Participants were randomly administered either a placebo (lactose) tablet or a 200 mg caffeine tablet (equal to 2–3 cups of coffee) at the beginning of each session 30 min before the experimental task. During this 30 min period participants processed the Edinburgh handedness inventory [56] and the chimeric faces test, a behavioral measure of cerebral lateralization of processing facial emotions [37], [59]. Unpaired Student’s t-tests revealed that the experimental groups did not differ in these measures (handedness: t(64) = 0.229, p = 0.766; chimeric faces: t(64) = −1,545, p = 0.127) and also not in their average usual coffee consumption (cups of coffee per day: t(64) = 0.130, p = 0.897).

The experiment consisted of two parts, a divided visual field lexical decision task and a subsequent arousal rating. Participants were seated 60 cm in front of 19′′ LCD monitor (resolution 1024*768 pixel, 60 Hz) with their head stabilized in a chin rest. Each trial started with a fixation cross in the center of the screen for 1000 ms. Stimuli were presented as uppercase letter strings for 150 ms to avoid eye-gaze refixations during stimulus presentation. All stimuli were randomly presented in either the right or left visual field followed by a ######## mask at the same position. The mask remained on screen until the button press or a maximum of 2850 ms, replaced by a blank screen before the next trial started with a new fixation cross. The center of the stimuli was subtended by a visual angle of 5 degrees (2–8 degrees) relative to the vertical midline. Pseudorandomization was used to assure that no more than three stimuli of the same condition (emotion, hemifield, lexicality) appeared subsequently in a row and that all conditions were presented with equal probability in each visual hemifield in a randomized order. Participants were instructed to decide as fast and accurately as possible whether the shortly presented letter string was an orthographically legal German word or not. Participants pressed the left mouse button if the string was a word and the right mouse button if it was not a word. To accommodate the participants with the task and the short presentation duration, 40 lexical decision training trials were presented before the actual stimulus set of 300 stimuli comprising 20 words and 20 pseudowords of 5 letters in length. This sample of 40 stimuli did not overlap with the experimental stimuli and the timing and hemifield presentation conditions were identical to that of the subsequent main experiment as described above. The main experiment was presented in one single block of 300 stimuli and lasted about 21 minutes. Immediately following the lexical decision task, participants were presented all words and the orthographically legal basewords of all pseudowords in a randomized order again with the instruction to rate the level of arousal associated with the words on a 7-point-Likert scale (1 =  calm to 7 =  highly arousing).

### Analyses

The data has been analyzed using R system for statistical computing (version 2.14.1, R Foundations for Statistical Computing) under the GNU General Public License (Version 2, June 1991). Outliers were defined as responses with a latency of more than 2.5 standard deviations from the average mean response latency of a subject and removed from all subsequent analyses (1.513% of all trials). Two different analyses were computed, a first analysis of variance procedure followed the signal detection approach described in [58]. Signal detection theory can be applied to examine two-choice decisions under conditions of uncertainty, and to derive measures of discrimination performance (i.e. perceptual sensitivity), and response bias, the response tendencies independent of discrimination performance [58], [60]. HITs were computed as the probability of a correct ‘word’ response and FA rates as the probability of incorrect ‘word’ responses. The performance measure P is then computed as the word-nonword discrimination performance (P = HIT-FA), and the response bias as B = FA/(1-P) [58], [61]. Performance measures and response biases per subject and condition were then subjected to a 2(HEMISPHERE: LVF, RVF) *3(EMOTION: positive, negative, neutral) repeated measures ANOVA with GROUP (caffeine, placebo) as the between subjects factor. In addition, to control for possible speed-accuracy trade-offs, a 4-way ANOVA (EMOTION, HEMISPHERE, LEXICALITY, GROUP) with log-transformed response latencies as the dependent variable was computed.

Because arousal measures were not controlled for in the nonwords, the analysis was repeated on error data at the single trial level. A linear mixed model (LMM) approach was used to examine the data using the lme4 package [62] as part of LanguageR [63] within the R statistical software. LMMs have the advantage over classical ANOVA approaches to incorporate independent, crossed subject and item random effects into the analyses when each unit of analysis is taken into account (instead of aggregated average measures per subject and condition). Thus, the LMM approach combines participants (F1) and items (F2) analyses into one coherent multiple regression model which additionally takes a random sampling of the participants into account to generalize the estimated effects to the population level. Despite these advantages of LMMs that have led to their widespread use in psycholinguistics in recent years (e.g. [63]), of particular interest for the present analysis is that, in an LMM, item information like the subjective ratings of arousal can easily be incorporated into the model to explain variance in the dependent variable.

Because error rates are binomial data, a generalized mixed effects model using a binomial link function and a mixed-effects logistic regression has been applied to the data [63] (see also [64] for a discussion of the advantages of generalized mixed effects models over classical ANOVA approaches in case of categorical outcome variables) with accuracy as the dependent variable and HEMISPHERE (LVF, RVF), EMOTION (positive, negative, neutral), LEXICALITY (word, pseudoword), and experimental GROUP as predictors, as well as all two-way interactions and the three-way interaction of EMOTION, HEMISPHERE and GROUP (see Material S1). Furthermore the lme4 package provides different goodness of fit measures that allow for model comparison between mixed effect models of different complexity (in number of estimated parameters). Thus, model comparison was applied to evaluate whether inclusion of subjective arousal and its two-way interactions with the other predictors was validated by the data (Table 2). For the final and best fitting model Wald’s z-values are reported, at a significance level that was set for all analyses at p = 0.05. Due to the short presentation durations and the presentation in the visual hemifields the overall error rate was rather high in the experiment so that eight participants data had to be discarded from the analyses in each group because of an unacceptable high error rate of > = 0.45.

**Table 2:** Results of Likelihood ratio tests comparing models w/o subjective arousal.

	Df	AIC	BIC	Chisq	Df	p-value
model A	19	17904	18048			
model B	20	17906	18058	0.0416	1	0.8385
model C	25	17905	18094	11.946	6	0.06332

AIC - Akaike Information Criterion, BIC - Bayesian information criterion, Df  =  Degrees of Freedom, Chisq  =  chi square value (−2*log-likelihood).

+model A - ERROR ∼ EMOTION +HEMISPHERE +GROUP +LEXICALITY +LEXICALITY*EMOTION.

+LEXICALITY*HEMISPHERE +LEXICALITY*GROUP +EMOTION*HEMISPHERE.

+EMOTION*GROUP +HEMISPHERE*GROUP.

+EMOTION*GROUP*HEMISPHERE +(1 | SUBJECT) +(1 | ITEM).

+model B - ERROR ∼ EMOTION +HEMISPHERE +GROUP +AROUSAL +LEXICALITY.

+LEXICALITY*EMOTION +LEXICALITY*HEMISPHERE +LEXICALITY*GROUP.

+EMOTION*HEMISPHERE +EMOTION *GROUP +HEMISPHERE*GROUP.

+EMOTION*GROUP*HEMISPHERE +(1 | SUBJECT) +(1 | ITEM).

+model C - ERROR ∼ EMOTION +HEMISPHERE +GROUP +AROUSAL +LEXICALITY.

+AROUSAL*GROUP +LEXICALITY*GROUP +LEXICALITY*HEMISPHERE.

+AROUSAL*HEMISPHERE +AROUSAL*EMOTION +LEXICALITY*EMOTION.

+EMOTION*HEMISPHERE +EMOTION*GROUP +HEMISPHERE*GROUP.

+AROUSAL*LEXICALITY +EMOTION*GROUP*HEMISPHERE.

+(1 | SUBJECT) +(1 | ITEM).

## Results

### Signal Detection Approaches

**Figure 1:**
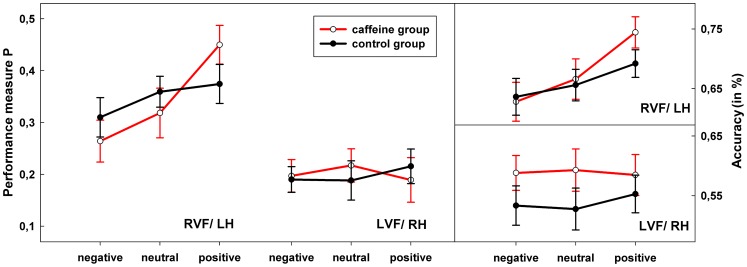
Average signal detection performance measures P and accuracy per condition and experimental group. Error bars represent the standard error of the mean. RVF/LH  =  right visual field/left hemisphere, LVF/RH  =  left visual field/right hemisphere.

The repeated measures ANOVA on the performance measure P revealed significant main effects of EMOTION (F(2,96) = 5.951, p = 0.003, η^2^ = 0.110) and HEMISPHERE (F(1,48) = 40.257, p<0.001, η^2^ = 0.456), and a significant EMOTION*HEMISPHERE interaction (F(2,96) = 7.012, p = 0.001, η^2^ = 0.127). No effect of GROUP and no two-way interaction with GROUP reached significance, but in accordance with our initial hypothesis a significant three-way EMOTION*HEMISPHERE*GROUP interaction was observed (F(2,96) = 4.539, p = 0.013, η^2^ = 0.086). To further examine this three-way interaction, the repeated measures ANOVA was split in two EMOTION*GROUP analyses in each hemisphere. While no significant result was visible in the RH, performance measure P revealed a significant emotion effect (F(2,96) = 14.859, p<0.001, η^2^ = 0.236) and a significant EMOTION*GROUP interaction in the LH (F(2,96) = 4.442, p = 0.014, η^2^ = 0.085). This interaction was due to a significant emotion effect in the caffeine group (F(2,48) = 16.320, p<0.001, η^2^ = 0405) in the LH that was not visible in the placebo control group (F(2,48) = 2.248, p = 0.117, η^2^ = 0.085). Follow-up pairwise comparisons show that the caffeine group emotion effect was based on higher performance measures when participants evaluated positive stimuli (positive - neutral, t(24) = 4.033, p<0.001; positive – negative, t(24) = 5.602, p<0.001; negative-neutral, t(24) = 1.572, p = 0.129). Figure 1 (left) depicts this small but significant interaction effect by showing that although performance to positive items was superior in the LH in both groups it is additionally enhanced in the caffeine group, a pattern that is not visible in the RH. This result pattern is even more pronounced in the accuracy data of word stimuli (Figure 1, right).

Regarding the bias measure B, the three-way repeated measures ANOVA only revealed a significant main effect of HEMISPHERE (F(2,96) = 5.577, p<0.05, η^2^ = 0.104) due to an overall greater response bias in the LVF/RH that indicates more liberal thresholds for ‘word’ responses independent of whether or not there actually was a word, i.e. a higher tendency to guess, when items are presented in the LVF(Table 3, Material S2). No sign of a speed-accuracy trade-off between the experimental groups could be observed as the ANOVA on log-transformed response latencies only revealed a significant effect of lexicality (words<pseudowords: F(1,14) = 26.206, p<0.001, η^2^ = 0.353). No further main effect, nor any interaction with GROUP reached significance (Material S3).

**Table 3:** Behavioral results. Average data per condition and group.

	HITs		False Alarms		Performance P		Bias B	
	caffeine	placebo	caffeine	placebo	caffeine	placebo	caffeine	placebo
**RVF/LH**								
** negative**	0.63(0.03)	0.64(0.03)	0.36(0.03)	0.33(0.02)	0.26(0.04)	0.31(0.04)	0.50(0.03)	0.52(0.03)
** neutral**	0.67(0.03)	0.66(0.03)	0.35(0.03)	0.30(0.02)	0.32(0.05)	0.36(0.03)	0.47(0.03)	0.53(0.03)
** positive**	0.74(0.03)	0.69(0.02)	0.29(0.03)	0.32(0.03)	0.45(0.04)	0.37(0.04)	0.48(0.05)	0.50(0.03)
**LVF/RH**								
** negative**	0.59(0.03)	0.53(0.03)	0.39(0.03)	0.34(0.03)	0.20(0.03)	0.19(0.02)	0.52(0.03)	0.58(0.03)
** neutral**	0.59(0.04)	0.53(0.04)	0.38(0.03)	0.34(0.03)	0.22(0.03)	0.19(0.04)	0.52(0.04)	0.58(0.04)
** positive**	0.58(0.03)	0.55(0.03)	0.40(0.04)	0.34(0.03)	0.19(0.04)	0.22(0.03)	0.51(0.03)	0.57(0.03)

RVF/LH  =  right visual field/left hemisphere, LVF/RH  =  left visual field/right hemisphere.

### Mixed-effect Logistic Regression

The mixed-effect logistic regression on the error data replicates the above pattern of results (see Material S1). Model comparison revealed that neither a model that includes subjective arousal and all two-way-interactions with subjective arousal (chi(6) = 11.94; p = 0.063) nor a model that only additionally includes subjective arousal (chi(1) = 0.04; p = 0.839) outperformed the model without subjective arousal and its interactions (see Table 2). Thus, inclusion of subjective arousal in the regression model did not significantly explain additional variance in the present analysis.

Thus, the best fitting model that contains EMOTION, HEMISPHERE, LEXICALITY and GROUP, and all two-way interactions of these and the EMOTION*HEMISPHERE*GROUP interactions, is characterized by a significant main effect of EMOTION (z =  −3.255, p = 0,001) due to fewer errors following positive items and a significant main effect of HEMISPHERE (z = 3.737, p<0,001) revealing that participants elicited fewer errors in the RVF/LH. Furthermore, HEMISPHERE significantly interacts with LEXICALITY (z =  −3.327, p = 0,001) and EMOTION (z =  −3.325, p = 0,001). The HEMISPHERE*LEXICALITY interaction effect is based on the fact that more errors are visible in the LVF/RH if participants processed words, whereas the HEMISPHERE*EMOTION effect was based on fewer errors to positive items in the RVF/LH. More important, significant interactions with GROUP were also obtained: a GROUP*LEXICALITY effect (z =  −5.177, p<0,001). Whereas the caffeine group made fewer errors in the word condition, this effect was reversed in the pseudoword condition where the control group showed fewer errors, i.e. the caffeine group more often falsely recognized pseudowords as words based on their correct phonology. Furthermore, a significant GROUP*EMOTION effect was visible (z = 2.292, p = 0,022) based on fewer errors to positive items observable in the caffeine group. Moreover the three-way interaction of EMOTION*HEMISPHERE*GROUP reached significance (z =  −2.112; p = 0.035), replicating the pattern of the signal detection ANOVA with an increase in accuracy for positive items in the left hemisphere (Material S1, also Figure 1).

## Discussion

Both analyses, the signal detection approach and the mixed-effect logistic regression agreed in the fact that caffeine affects performance in the affective lexical decision task. It is evident that caffeine does not affect lexical decision performance in general, but in accordance with the initial hypotheses specific interactions with the emotional valence of the stimulus were observed. Of particular interest in the present study is the three-way interaction between GROUP, EMOTION and HEMISPHERE (see Figure 1). Thus, the enhancement effect of caffeine in word recognition is restricted to a facilitated processing of positive items in the RVF/LH, an effect that was predicted by a combination of the left hemispheric advantage of positive stimuli as proposed by the VHM [30], [31] and an underlying dopaminergic system activity (triggered by caffeine intake) associated with the processing of positive information. This result points to a dopaminergic explanation of the left hemisphere advantage of positive stimuli in word processing. Further in line with this interpretation is the observed EMOTION*GROUP effect of fewer errors to positive items in the caffeine group, but the specificity and the direction of the three-way interaction support a localization of the positivity advantage in the language-dominant left hemisphere.

In general the present results mirror that of previous divided visual field emotional word recognition studies. Word recognition was superior in the RVF/LH compared to the LVF/RH, and the recognition of positive words was superior to that of negative words (e.g. [28]). Moreover a significant interaction between HEMISPHERE and EMOTION was visible: Whereas no emotion effect reached significance in the LVF/RH, the positivity advantage with facilitated processing of positive compared to negative items was visible in the left hemisphere. Thus, the emotional word recognition effect in the present study should be attributed to processing of positive verbal information in the left hemisphere. Such an effect is consistent with the predictions of the VHM [30]–[32].

Abbassi and colleagues [65] further proposed that emotional information is automatically activated when processed by the left hemisphere. Given that the target stimuli were presented for 150 ms in this study, the observed differences between left and right hemispheric processing seem consistent with the assumption of an early locus of this effect in the word recognition stream. Of note is that an early automatic evaluation has been discussed to be involved in emotional word recognition [13], [65] and effects prior to 150 ms have repeatedly been observed [14], [20]. Moreover, Hofmann and colleagues were able to localize an emotional word recognition effect in a left-hemispheric posterior temporal brain region [14] discussed to support visual word form processing.

Still, the link between the dopaminergic system and the posterior temporal lobe seems rather unspecific. On the other hand, it is well known that the striatum is activated during word recognition [13], [66], [67] and more generally in perceptual decision making [68]. The striatum has been discussed to be related to response criterion setting [69]. It seems likely that the facilitated processing of positive words also affects the setting of a trial-by-trial response criterion, which could describe a mechanism that explains how these two streams of processing, dopaminergically driven decision making and emotional word recognition, interact in the lexical decision task. Also, a LH striatal dominance is known and has been linked to motor lateralization and right hand preference [70], [71]. Thus, a possible neural mechanism how caffeine affects emotional word recognition would link increased dopaminergic transmission following caffeine consumption in the striatum [42] to LH dominant activations in right-handers [41], [42], [70]. Thus the availability of dopamine that is itself closely tied to motor preparedness [72] may specifically interact with LH activations in the basal ganglia in language processing [73], [74] and striatal activations when recognizing emotional positive words. Future neuroimaging studies are needed to examine this possible link.

Both analyses revealed a GROUP*EMOTION interaction (at least in left-hemispheric processing) which undermines the emotional effects in the present results. Thus, emotion effects in the placebo control group are diminished – which is surprising given the results of previous divided visual field emotional word recognition studies [28], [58] and the generally known effects of emotional content in word recognition [12], [13], [22]. Of note is, that overall the error rates were high, which is indicative of a high task difficulty of the present procedure that could have contributed to these small effects. Moreover, no emotion effects were observed in the RH (see Figure 1) and also not in the response latencies, which is at odds with Holtegraves and Felton’ study [28]. We would like to address this to methodological differences between these two studies, in particular the focus on response latencies in [28] vs. a signal-detection paradigm in the present study with a focus on accuracy, as well as the use of short stimulus presentation times and well controlled low-arousing word material).

Stimulus’ arousal is also discussed to modulate emotional word recognition effects [14], [22]. By computing a mixed-effect logistic regression on the error data, we were able to estimate the effect of subjectively judged stimulus arousal in explaining the present results. Surprisingly, a regression model that contained stimulus arousal and its interactions with the other variables, was not superior to a model without arousal, which leads to the assumption that the effects of stimulus’ arousal were small in the present study.

Alternatively, the control of caffeine intake itself could have contributed to these small emotion effects in the placebo group, i.e. previous divided visual field studies did not ask their participants to refrain from caffeine or nicotine in 12 hours in advance [28], [58]. Thus, for example, caffeine mood effects have been interpreted under the withdrawal reversal hypothesis [54], [75]: Due to the fact that studies mainly examined caffeine effects after 12 hours of abstinence, it is discussed whether the positive effects of caffeine result from the removal of withdrawal effects in normal caffeine consumers. Accordingly, compared to the placebo group results, an effect of everyday caffeine consumption could have contributed to these earlier results, which should also be addressed in future examinations.

To what extend did early and late effects of word recognition contribute to the present results? The HEMISPHERE*LEXICALITY interaction indicates possible differences in the recognition of words and pseudowords. Words led to more errors in the LVF/RH (0.44) compared to pseudowords (0.36) whereas accuracy in the RVF/LH was comparable in both conditions (0.33/0.32). Pseudowords receive their meaning from their phonology, which models of word recognition, like the MROM-p [76], assume to rely on late and effortful processing in visual word recognition. Such models propose that at sublexical processing stages, orthographic information has to be transferred to sublexical phonological codes, which activate lexical entries at the phonological word level [76] that trigger the button press. In contrast, words are processed directly along the associations between sublexical and lexical orthographic.

This observed advantage of pseudowords in the LVF/RH fits the proposed RH superiority at post-lexical processing stages [58]. Together with the model predictions it seems likely that late phonological effects modulated the responses to RH stimuli, whereas decisions to LH stimuli, in accordance with its function in visual verbal processing relied more on early (orthographic) processing. If this interpretation is true, the GROUP*LEXICALITY interaction with more errors in the caffeine group to pseudowords would also locate the caffeine effect at a late processing stage when phonology is processed. A pseudoword, that is per definition word-like based on its phonology, is harder to correctly reject when its phonology is already being processed. This is exactly what is visible in the caffeine group. Thus, based on this effect, it is also possible that caffeine has a late effect on the processing of visual verbal material, probably associated with the decision stage. This would also be more consistent with the role of dopaminergic transmission in the striatum in perceptual decision making and the lexical decision task [68], [69], but cannot be solved based on the present results.

### Conclusion

The application of caffeine in the experimental group resulted in small but significant effects compared to a placebo control group, which reveal that caffeine does not simply affect overall task performance in a simple two-choice decision paradigm. Presenting emotional words and nonwords in a divided visual field paradigm led to a higher order interaction between the emotional valence of the stimuli, their initial hemispheric processing and group membership. This interaction with an enhanced processing of positive stimuli after caffeine intake is consistent with the initial hypothesis of a dopaminergically driven positivity advantage in emotional word recognition that seems specifically boosted in the language-dominant left brain when processing verbal stimuli. A comparable effect when processing negative or emotionally arousing words and pseudowords was not observed. This pattern additionally underlines the differential effects of positive and negative valence in emotional word recognition.

## Supporting Information

Material S1 **Result Table of the Mixed-effects logistic regression with accuracy as the dependent variable.**
(DOC)

Material S2 **Result Tables of all 3-way within subjects ANOVA of EMOTION, HEMISPHERE and GROUP.**
(DOC)

Material S3 **Result Table of the 4-way within subjects ANOVA of EMOTION, HEMISPHERE, LEXICALITY and GROUP.**
(DOC)
