# Supplementary material for: Caffeine Improves Left Hemisphere Processing of Positive Words
Source: PLoS One. 2012 Nov 7;7(11):e48487. doi: 10.1371/journal.pone.0048487 (PMC3492460; doi:10.1371/journal.pone.0048487)
Supplement: Material S1 — Result Table of the Mixed-effects logistic regression with accuracy as the dependent variable. (DOC) [file pone.0048487.s001.doc]

**S1 Result Table of the Mixed-effects logistic regression** with accuracy as the dependent variable. Please note that the reference condition is EMOTION_neg_ *HEMISPHERE_RVF/LH_ *GROUP_caffeine_. The estimates indicate the direction of an effect. The table can be read as effects relative to the reference condition, i.e. the effect of positive EMOTION (EMOTION_pos_) of -0.529 indicates that the error rate decreases by 0.529 if a stimulus is positive (compared to the effect of negative stimuli) (see [65]).

model A - ERROR ~ EMOTION +HEMISPHERE +GROUP +LEXICALITY +LEXICALITY*EMOTION

+LEXICALITY*HEMISPHERE +LEXICALITY*GROUP +EMOTION*HEMISPHERE

+EMOTION*GROUP +HEMISPHERE*GROUP

+EMOTION*GROUP*HEMISPHERE +(1 | SUBJECT) +(1 | ITEM)

| **Fixed effects:** | **Estimate(SE)** | **Walds z -value** | **Pr(>|z|)** |
| --- | --- | --- | --- |
| (Intercept) | -0.705(0.131) | -5.382 | **<0.001** |
| EMOTION_neu_ | -0.119(0.160) | -0.745 | 0.456 |
| EMOTION_pos_ | -0.529(0.162) | -3.255 | **0.001** |
| HEMISPHERE_LVF/RH_ | 0.407(0.109) | 3.737 | **<0.001** |
| GROUP_control_ | 0.096(0.127) | 0.752 | 0.452 |
| LEXICALITY_pseudo_ | 0.093(0.148) | 0.629 | 0.529 |
| EMOTION_neu_ *LEXICALITY_pseudo_ | 0.010(0.193) | 0.050 | 0.960 |
| EMOTION_pos_ *LEXICALITY_pseudo_ | 0.142(0.193) | 0.737 | 0.461 |
| HEMISPHERE_LVF/RH_ *LEXICALITY_pseudo_ | -0.303(0.091) | -3.327 | **<0.001** |
| GROUP_control_ *LEXICALITY_pseudo_ | -0.376(0.073) | -5.177 | **<0.001** |
| EMOTION_neu_ *HEMISPHERE_LVF/RH_ | 0.071(0.141) | 0.504 | 0.614 |
| EMOTION_pos_ *HEMISPHERE_LVF/RH_ | 0.472(0.142) | 3.325 | **<0.001** |
| EMOTION_neu_ *GROUP_control_ | 0.021(0.128) | 0.164 | 0.869 |
| EMOTION_pos_ *GROUP_control_ | 0.298(0.130) | 2.292 | **0.022** |
| HEMISPHERE_LVF/RH_ *GROUP_control_ | 0.132(0.125) | 1.052 | 0.293 |
| EMOTION_neu_ *HEMISPHERE_LVF/RH_ *GROUP_control_ | -0.008(0.177) | -0.044 | 0.965 |
| EMOTION_pos_ *HEMISPHERE_LVF/RH_ *GROUP_control_ | -0.378(0.179) | -2.112 | **0.035** |

neu = neutral, pos = positive, neg = negative, RVF/LH = right visual field/ left hemisphere, LVF/RH = left visual field/ right hemisphere, pseudo = pseudoword
