# Supplementary material for: Caffeine Improves Left Hemisphere Processing of Positive Words
Source: PLoS One. 2012 Nov 7;7(11):e48487. doi: 10.1371/journal.pone.0048487 (PMC3492460; doi:10.1371/journal.pone.0048487)
Supplement: Material S2 — Result Tables of all 3-way within subjects ANOVA of EMOTION, HEMISPHERE and GROUP. (DOC) [file pone.0048487.s002.doc]

**S2 Result Tables of all 3-way within subjects ANOVA of EMOTION, HEMISPHERE and GROUP** on signal detection theory performance measure P and bias measure B as dependent variables, and subsequent follow-up ANOVAs

Table S2-1: overall 3-way ANOVA

| **Effect** | **Df** | F-value | Pr(>F) |
| --- | --- | --- | --- |
| GROUP | 1,48 | 0 | 0.993 |
| EMOTION | 2,96 | 5.951 | **0.004** |
| HEMISPHERE | 1,48 | 40.275 | **<0.001** |
| GROUP*EMOTION | 2,96 | 0.675 | 0.512 |
| GROUP*HEMISPHERE | 1,48 | 0.023 | 0.880 |
| EMOTION*HEMISPHERE | 2,96 | 7.012 | **0.001** |
| GROUP*EMOTION*HEMISPHERE | 2,96 | 4.539 | **0.013** |

Dependent variable is Performance P

**Table S2-2: 2-way ANOVA** (RVF/LH only)

| **Effect** | **Df** | F-value | Pr(>F) |
| --- | --- | --- | --- |
| GROUP | 1,48 | 0.006 | 0.937 |
| EMOTION | 2,96 | 14.859 | **<0.001** |
| GROUP*EMOTION | 2,96 | 4.442 | **0.014** |

Dependent variable is Performance P

**Table S2-3: 2-way ANOVA** (LVF/RH only)

| **Effect** | **Df** | F-value | Pr(>F) |
| --- | --- | --- | --- |
| GROUP | 1,48 | 0.007 | 0.932 |
| EMOTION | 2,96 | 0.075 | 0.928 |
| GROUP*EMOTION | 2,96 | 0.542 | 0.584 |

Dependent variable is Performance P

**Table S2-4: 1-way ANOVA** (RVF/LH only, caffeine group)

| **Effect** | **Df** | F-value | Pr(>F) |
| --- | --- | --- | --- |
| EMOTION | 2,48 | 16.32 | **<0.001** |

Dependent variable is Performance P

**Table S2-5: 1-way ANOVA** (RVF/LH only, placebo control group)

| **Effect** | **Df** | F-value | Pr(>F) |
| --- | --- | --- | --- |
| EMOTION | 2,48 | 2.248 | 0.117 |

Dependent variable is Performance P

Table S2-6: overall 3-way ANOVA

| **Effect** | **Df** | F-value | Pr(>F) |
| --- | --- | --- | --- |
| GROUP | 1,48 | 1.586 | 0.214 |
| EMOTION | 2,96 | 0.3910.677 | 0.677 |
| HEMISPHERE | 1,48 | 5.577 | **0.022** |
| GROUP*EMOTION | 2,96 | 0.338 | 0.714 |
| GROUP*HEMISPHERE | 1,48 | 0.372 | 0.5449 |
| EMOTION*HEMISPHERE | 2,96 | 0.113 | 0.893 |
| GROUP*EMOTION*HEMISPHERE | 2,96 | 0.244 | 0.784 |

Dependent variable is Bias B
