# Supplementary material for: Caffeine Improves Left Hemisphere Processing of Positive Words
Source: PLoS One. 2012 Nov 7;7(11):e48487. doi: 10.1371/journal.pone.0048487 (PMC3492460; doi:10.1371/journal.pone.0048487)
Supplement: Material S3 — Result Table of the 4-way within subjects ANOVA of EMOTION, HEMISPHERE, LEXICALITY and GROUP. (DOC) [file pone.0048487.s003.doc]

**S3 Result Table of the 4-way within subjects ANOVA of EMOTION, HEMISPHERE, LEXICALITY and GROUP** on (log-transformed) response latencies

| **Effect** | **Df** | F-value | Pr(>F) |
| --- | --- | --- | --- |
| GROUP | 1,48 | 1.419 | 0.239 |
| EMOTION | 2,96 | 2.222 | 0.114 |
| HEMISPHERE | 1,48 | 0.445 | 0.508 |
| LEXICALITY | 1,48 | 26.206 | **<0.001** |
| GROUP*EMOTION | 2,96 | 0.027 | 0.974 |
| GROUP*HEMISPHERE | 1,48 | 1.506 | 0.226 |
| GROUP*LEXICALITY | 1,48 | 3.061 | 0.08/ |
| EMOTION*HEMISPHERE | 2,96 | 0.740 | 0.480 |
| EMOTION*LEXICALITY | 2,96 | 2.482 | 0.089 |
| HEMISPHERE*LEXICALITY | 1,48 | 1.443 | 0.236 |
| GROUP*EMOTION*HEMISPHERE | 2,96 | 0.991 | 0.375 |
| GROUP*EMOTION*LEXICALITY | 2,96 | 1.470 | 0.235 |
| GROUP*HEMISPHERE*LEXICALITY | 1,48 | 0.114 | 0.737 |
| EMOTION*HEMISPHERE *LEXICALITY | 2,96 | 0.734 | 0.483 |
| GROUP*EMOTION*HEMISPHERE* LEXICALITY | 2,96 | 0.045 | 0.956 |
